# Chinese Herbal Medicine Fuzheng Kang-Ai Decoction Inhibited Lung Cancer Cell Growth through AMPK*α*-Mediated Induction and Interplay of IGFBP1 and FOXO3a

**DOI:** 10.1155/2016/5060757

**Published:** 2016-02-04

**Authors:** Fang Zheng, Jingjing Wu, Xiong Li, Qing Tang, LiJun Yang, Xiaobing Yang, WanYin Wu, Swei Sunny Hann

**Affiliations:** ^1^Laboratory of Tumor Biology and Target Therapy, Guangdong Provincial Hospital of Chinese Medicine, The Second Clinical Medical College, University of Guangzhou Traditional Chinese Medicine, Guangzhou, Guangdong Province 510120, China; ^2^Central Laboratory, Guangdong Provincial Hospital of Chinese Medicine, The Second Clinical Medical College, University of Guangzhou Traditional Chinese Medicine, Guangzhou, Guangdong Province 510120, China; ^3^Department of Medical Oncology, Guangdong Provincial Hospital of Chinese Medicine, The Second Clinical Medical College, University of Guangzhou Traditional Chinese Medicine, Guangzhou, Guangdong Province 510120, China; ^4^Higher Education Mega Center, No. 55, Neihuan West Road, Panyu District, Guangzhou, Guangdong Province 510006, China

## Abstract

The aim of this study is to investigate the actions of Chinese herbal medicine, called “Fuzheng Kang-Ai” (FZKA for short) decoction, against non-small cell lung cancer (NSCLC) and its mechanisms* in vitro* and* in vivo*. We showed that the effect of FZKA decoction significantly inhibited growth of A549 and PC9 cells. Furthermore, FZKA increased phosphorylation of AMP-activated protein kinase alpha (AMPK*α*) and induced protein expression of insulin-like growth factor (IGF) binding protein 1 (IGFBP1) and forkhead homeobox type O3a (FOXO3a). The specific inhibitor of AMPK*α* (Compound C) blocked FZKA-induced protein expression of IGFBP1 and FOXO3a. Interestingly, silencing of IGFBP1 and FOXO3a overcame the inhibitory effect of FZKA on cell growth. Moreover, silencing of IGFBP1 attenuated the effect of FZKA decoction on FOXO3a expression, and exogenous expression of FOXO3a enhanced the FZKA-stimulated phosphorylation of AMPK*α*. Accordingly, FZKA inhibited the tumor growth in xenograft nude mice model. Collectively, our results show that FZKA decoction inhibits proliferation of NSCLC cells through activation of AMPK*α*, followed by induction of IGFBP1 and FOXO3a proteins. Exogenous expression of FOXO3a feedback enhances FZKA decoction-stimulated IGFBP1 expression and phosphorylation of AMPK*α*. The reciprocal interplay of IGFBP1 and FOXO3a contribute to the overall responses of FAKA decoction.

## 1. Introduction

Lung cancer has become the leading cause of cancer deaths in both men and women [1]. Non-small cell lung cancer (NSCLC) accounts for around 80% of lung cancer cases [2]. In spite of the advancement in the therapeutic approaches, the outcome remains unchanged, resulting in poor quality of life (QOL) and survival. A large body of evidence demonstrated promising outcome of other nonsurgical treatments in patients with advanced lung cancer. Traditional Chinese medicine (TCM) including Chinese herbal medicine (CHM) has increasingly become popular treatment strategies in cancer patients. TCM can alleviate the clinical symptoms and treatment related complications, improve the QOL, and reduce the side effects of conventional treatment in several cancer types [3–5]. CHM, called “Fuzheng Kang-Ai” (FZKA for short) decoction, prescribed by Dr. WanYin Wu, has been used to treat NSCLC in Guangdong Provincial Hospital of TCM for over a decade and showed to enhance the disease control rate (DCR) and time to progression (TTP) in patients with advanced NSCLC [6]. Also, in a recent study, we found that treatment with epidermal growth factor receptor-tyrosine kinase inhibitor (EGFR-TKI), gefitinib, with FZKA decoction prolonged the progression-free survival (PFS) or median survival time (MST) in patients with NSCLC as compared to that with gefitinib alone [7]. These suggested the potential therapeutic beneficial effects of FZKA decoction in lung cancer treatment. However, the detailed molecular mechanism underlining this has not been fully elucidated.

Insulin-like growth factor (IGF) binding protein 1 (IGFBP1) is produced primarily by maternal deciduas and fetal organs [8, 9] and functioned in the cellular level to regulate cell growth and survival [10]. IGFBP-1 interacts with several other proteins in addition to ligands IGF-I and IGF-II and plays an important role in the development and progression of cancer [10–13]. Animal studies also demonstrated the inhibitory effect of IGFBP1 in tumor growth [14]. However, opposite results were also reported [15]. Thus, IGFBP-1 may have dual effects on cancer progression depending upon the environment contexts. We believed that the biological function of IGFBP-1 becomes more complicated than we previously thought.

Forkhead homeobox type O3a (FOXO3a, FKHRL1) belongs to the family of mammalian forkhead transcription factors implicated in the regulation of variety of biological functions, such as cell cycle, DNA repair, and cell differentiation [16], and is considered a potential target for therapeutic strategies against cancers [17]. During tumor development, inhibition of FOXO3a stimulated cell transformation and tumor progression [18]. On the contrary, overexpressed FOXO3a suppressed growth and induced apoptosis in cancer cells [19–21].

In this study, we explored the potential mechanism by which FZKA inhibits growth of NSCLC cells.

## 2. Materials and Methods

### 2.1. Chinese Herbal Medicine (CHM)

The CHM FZKA decoction prescribed by Dr. WanYin Wu in Guangdong Provincial Hospital of Traditional Chinese Medicine was provided by Guangdong Kangmei Pharmaceutical Company Ltd. (Guangdong, China). The primary composition has been reported previously [7], which consisted of* Pseudostellaria heterophylla* (Miq.) 30 g,* Atractylodes macrocephala* Koidz. 15 g,* Astragalus membranaceus* Bge. 30 g,* Oldenlandia diffusa* Roxb. 30 g,* Solanum nigrum* L. 30 g,* Salvia chinensis* Benth 30 g,* Cremastra appendiculata* Makino 30 g,* Coix lachrymal-jobi* L. 30 g,* Akebia quinata* Decne 30 g,* Rubus parviflolius* L. 30 g,* Curcuma kwangsiensis* S.G. 15 g, and* Glycyrrhiza uralensis* Fisch. 10 g. The calculated clinical dosages are based on the concentrations in the total crude drug (g) over TCM decoction volume, rather than the actual water concentration. For FZKA decoction, that is a total of 310 g/day crude drug and about 250 mL after boiled herbal decoction, 310 g/250 mL = 1.24 g/mL. The preparation method of FZKA decoction was as follows: the herbs (310 g) were soaked for 30 min and then reflux-extracted with 1 L water twice for 1 h. The aqueous extract was filtered and concentrated using a rotary evaporator, and we made the final stock concentration of 3.1 g/mL. Afterwards, the resulting supernatant was diluted in the cell culture media to 100 mg/mL and then was filtered (0.2 *μ*M) to become the sterilized stock solution as described by another study [22].

### 2.2. High Performance Liquid Chromatography (HPLC) Analysis

We performed the initial batch to batch consistency studies using high performance liquid chromatography (HPLC). Briefly, the samples solutions were put into the HPLC system (Agilent 1200 HPLC system, Santa Clara, CA, USA) and then were separated on the chromatographic column C_18_ (250 × 4.6 mm, 5 *μ*m, ACE, Scotland). The mobile phase consisted of deionized water with 0.1% formic acid (A) and acetonitrile with 0.1% formic acid (B). The gradient elution program was as follows: 5% B at 0–5 min, 5–20% B at 5–10 min, 20–40% B at 10–15 min, 40–95% B at 15–40 min, and 95–100% B at 40–45 min. The flow rate was 1.0 mL/min, and the detection wavelength was set at 280 nm. The injection volume was 10 *μ*L and the column temperature was maintained at 30°C. In addition, the chemical profiling of main constituents in FZKA decoction using ultra-high pressure liquid chromatography coupled with LTQ Orbitrap mass spectrometry was performed (see supplementary data for the details in Supplementary Material available online at http://dx.doi.org/10.1155/2016/5060757).

### 2.3. Reagents and Cell Culture

MTT powder and Compound C (a special inhibitor of AMPK) were purchased from Sigma Aldrich (St. Louis, MO, USA). Monoclonal antibodies specific of FOXO3a, total AMPK*α*, and the phosphor-forms were purchased from Cell Signaling Technology Inc. (Beverly, MA, USA). The IGFBP1 antibody was obtained from Abcam (Cambridge, MA, USA). IGFBP1 and FOXO3a small interfering RNAs (siRNAs) and Lipofectamine RNAiMAX Transfection Reagent were ordered from Life Technologies (Grand Island, NY, USA). The FOXO3a-GFP and N1-GFP plasmids were kindly provided by Dr. Frank M. J. Jacobs (Rudolf Magnus Institute of Neuroscience, University Medical Center, Utrecht, the Netherlands) and were reported previously [23]. The NSCLC cell lines (A549, PC9) were purchased from Cell Bank, Type Culture Collection, Chinese Academy of Sciences (Shanghai, China), and grown in RPMI-1640 medium supplemented with 10% (v/v) FBS, 100 U/mL streptomycin, and 100 U/mL penicillin (Gibco, Beijing, China). In addition, the medium of A549-luc was added to Geneticin (G-418) Sulfate (Life Technologies, Carlsbad, CA, USA) at concentration of 200 *μ*g/mL. When cells reached 80–90% confluence, they were digested with 0.25% trypsin for passage for the following experiments.

### 2.4. Cell Viability Assay

Cell viability was measured using the 3-(4, 5-dimethylthiazol-2-yl)-2, 5-diphenyltetrazolium bromide (MTT) assay reported previously [21]. NSCLC cells were seeded in 96-well plates at 5 × 10^3^ cells/well and incubated at 37°C in complete medium for 24 h before being treated with increasing concentrations of FZKA decoction for up to 72 h. In separated experiments, NSCLC cells were transfected with control, IGFBP1, or FOXO3a siRNAs (50 nM) for 24 h before exposure of the cells to FZKA decoction for an additional 24 h. Afterwards, cell viability was measured using MTT assay according to the instruction from the provider. Absorbance at 570 nm was determined through the use of ELISA reader (Perkin Elmer, Victor X5, Waltham, MA, USA).

### 2.5. Gene Microarray Analysis

NimbleGen Gene chip microarray analysis was conducted on NSCLC A549 cells at CapitalBio Corporation according to the instruction from the provider (Beijing, China). Briefly, after being treated with FZKA decoction for 24 h, total RNA was extracted using Trizol reagent (Invitrogen, Carlsbad, CA, USA). Array hybridization, washing, and scanning were carried out according to the NimbleGen's Expression user's guide. Subsequently, the arrays were scanned with a Roche-NimbleGen MS200 confocal laser scanner (Roche NimbleGen, Inc., Madison, WI, USA), and the obtained images were analyzed using NimbleScan Molecular Annotation System 3.0. The ratio of the two fluorescent signal intensities at each spot on the microarray is commonly used to infer the relative amounts of DNA levels. We employed the NimbleScan 2.6 image analysis software for standardization and normalization of microarray data. For the differentially expressed gene (DEG) detection, statistical *t*-tests with multiple test correction using methods such as Robust Multichip Analysis (RMA) were performed with the threshold of expressed genes set at a false discovery rate (FDR) of 0.01–0.05. Up- or downregulation of DEGs was determined as fold changes.

### 2.6. Quantitative Real-Time PCR

A quantitative real-time PCR (qRT-PCR) assay was developed for the detection and quantification of IGFBP1 transcripts using GAPDH as an endogenous control. The primers used in this study were designed as follows: IGFBP1 forward 5′-TCACAGCAGACAGTGTGAGAC-3′; reverse 5′-CCCAGGGATCCTCTTCCCAT-3′; GAPDH forward 5′-AAGCCTGCCGGTGACTAAC-3′; reverse 5′-GCGCCCAATACGACCAAATC-3′. qRT-PCR was performed in a 20 *μ*L mixture containing 2 *μ*L of the cDNA preparation, 10 *μ*L 2x SYBR Green Premix ExTaq (Takara), and 10 *μ*M primer on an ABI 7500 Real-Time PCR System (Applied Biosystems, Grand Island, NY, USA). The PCR conditions were as follows: 10 min at 95°C, followed by 40 cycles of 15 s at 95°C, and 1 min at 60°C. Each sample was tested in triplicate. Threshold values were determined for each sample/primer pair, and the average and standard errors were calculated.

### 2.7. Western Blot Analysis

Briefly, NSCLC cells were harvested, washed, and lysed with 1 × RAPI buffer. Protein concentrations were determined by the Thermo BCA protein assay Kit. Equal amounts of protein from cell lysates were solubilized in 5 × SDS sample buffer and separated on 10% SDS polyacrylamide gels and transferred onto polyvinylidene fluoride membranes. Membranes were blocked with 5% nonfat milk in TBST and incubated with primary antibodies against AMPK*α* and their phosphor-forms, IGFBP1 and FOXO3a at 4°C overnight. Afterwards, the membranes were washed and incubated with a secondary antibody against rabbit IgG for 1 h, followed by washing and transferring into ECL solution (Millipore, Darmstadt, Germany), and exposed and scanned under the Bio-Rad ChemiDoc XRS+ Chemiluminescence imaging system (Bio-Rad, Hercules, CA, USA).

### 2.8. Treatment with IGFBP1 and FOXO3a siRNAs

The detailed procedure was reported previously [21]. In brief, cells were seeded in 6-well or 96-well culture plates in RPMI 1640 medium containing 10% FBS (no antibodies), grown up to 50–70% confluence before incubation with siRNAs. IGFBP1 or FOXO3a and control siRNAs (up to 25 nM each) were transfected using the Lipofectamine RNAiMAX reagent according to the manufacturer's instructions. After culturing for up to 24 h, the cells were washed and resuspended in fresh media in the presence or absence of FZKA decoction for an additional 24 h for all other experiments.

### 2.9. Electroporated Transfection Assays

The detailed procedure was reported previously [21]. NSCLC cells (1 × 10^7^ cells/mL) were washed and centrifuged, followed by removing the medium. Afterwards, the cells in the tubes were added to RPMI 1640 medium containing 10% FBS (no antibodies). After resuspending the cells, 5 *μ*g N1-GFP or FOXO3a-GFP plasmid DNA was added for 200 *μ*L cell suspension and the electroporation plates were put in the MXcell plate chamber and closed the lid in Gene Pulser II Electroporation System (Bio-Rad, Hercules, CA, USA). The electroporation conditions on the plates to deliver 150 V/5 ms square wave were adjusted until reaching the optimum. After electroporation was completed, the cells were transferred to a tissue culture plate. Cells were incubated for 24 h at 37°C and then treated with FZKA decoction for an additional 24 h.

### 2.10. Animal Studies

All* in vivo* procedures were approved by Institutional Animal Care and Use Committee of Guangzhou University of Chinese Medicine (the Ethics Approval Number 2014013). A total of 42 eight-week-old female nude mice were obtained from Guangdong Provincial Research Center for Laboratory Animal Medicine (Foshan, Guangdong, China) and were maintained at the Animal Center of Guangdong Provincial Hospital of Chinese Medicine in a specific pathogen-free environment with food and water provided. A549 cells carrying luciferase report gene (A549-Luc) (1.5 × 10^6^ cells) in 100 *μ*L PBS were injected subcutaneously into the flank region of female nude mice. Xenografts were allowed to grow for over one week when the initial measurement was made with calipers. Mice were randomly divided into control and FZKA treatment groups (0.4 mL per mouse in each treatment via gavage, based on the 62 g/kg, 20 g average mouse weight), for 30 days (*n* = 6/group). The calculated formula between human and mouse according to the body surface area is mouse dose (g/kg) = human dose (g/kg) × 3/37 [24]. For bioluminescence imaging (BLI) procedure, mice were anesthetized by inhalation of 2% isoflurane. Each set of mice was injected intraperitoneally with 150 mg/kg D-luciferin (Caliper; PerkinElmer, Waltham, MA, USA) in approximately 200 *μ*L. The intensity of BLI signal was determined using the IVIS-200 Imaging System (Xenogen, Alameda, CA, USA). Quantification of bioluminescence was reported as photons/sec. The body weights of the mice were measured once a week. All mice were euthanized on day 30, and tumors were removed and weighted. Tumor volume measurements were calculated using the formula for an oblong sphere: volume = (width^2^ × length).

### 2.11. Statistical Analysis

All data were expressed as mean ± SD of three independent experiments. Differences between groups were assessed by one-way ANOVA and significance of difference between particular treatment groups was analyzed by Tukey's Multiple Comparison Test for multiple comparison involved (GraphPad Prism 5.0 software, La Jolla, CA, USA). Values were considered significant if *p* < 0.05.

## 3. Results

### 3.1. Chromatogram of Four Batches of FZKA Decoction by HPLC

**Figure 1:**
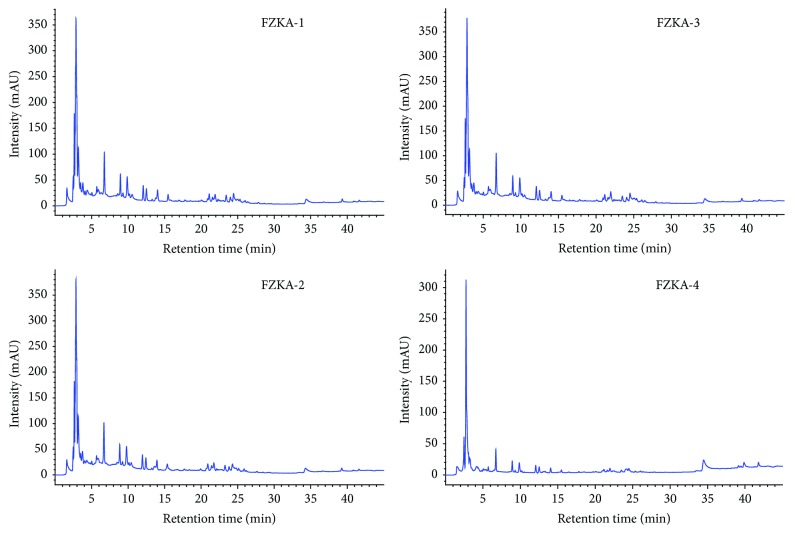
The chromatograms in FZKA decoction by HPLC method. The water extraction of the compound prescriptions of the different groups of the FZKA decoction was qualitatively analyzed by HPLC method as described in Section 2. Conditions: column: C_18_ column (250 × 4.6 mm, 5 *μ*m); mobile phase: deionized water with 0.1% formic acid (Solvent A) and acetonitrile with 0.1% formic acid (Solvent B). Flow rate: 1.0 mL/min; column temperature: 30°C; injection volume: 10 *μ*L. Four batches of FZKA decoction water extracts including a mixture of three different batches (FZKA1–4) are presented separately.

The HPLC method was validated by defining the method parameters including identity confirmation, accuracy, stability, and recovery. Results showed that the HPLC chromatograms in different drinks of FZKA decoction had similar pattern (Figure 1). This result indicated that the main components in the different batches (drinks) of FZKA decoction showed the similar pattern in chromatograms by HPLC method. Further studies using qualitative and quantitative analysis are required to demonstrate the batch to batch consistency.

### 3.2. FZKA Decoction Inhibited Growth of Human NSCLC Cells

**Figure 2:**
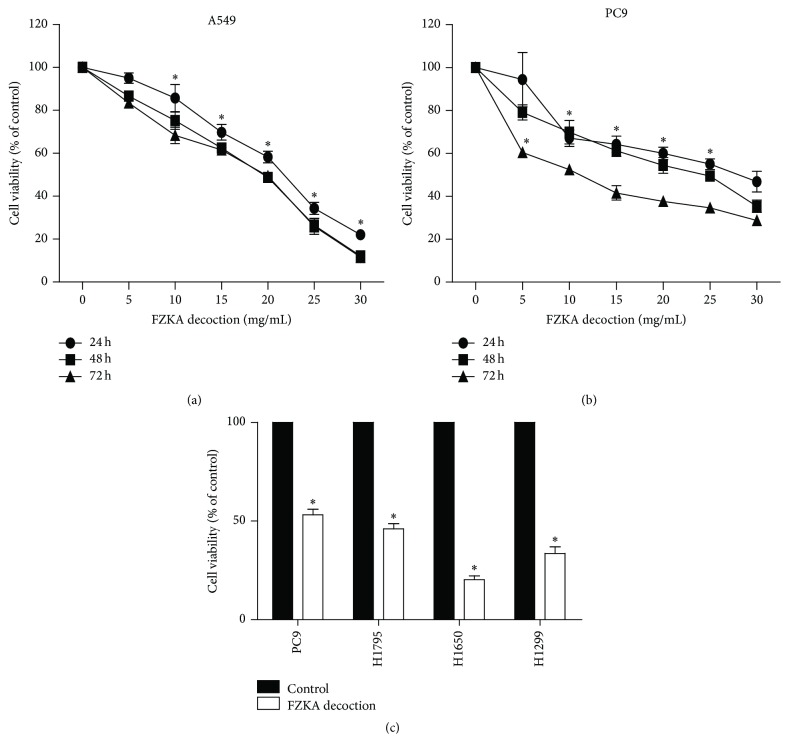
FZKA decoction inhibited growth of human NSCLC cells in time- and dose-dependent manner. (a-b) A549 (a) and PC9 (b) cells were treated with increased concentrations of FZKA decoction for up to 72 h to examine the cell viability. (c) NSCLC cell lines indicated were treated with FZKA decoction (20 mg/mL) for 48 h. The cell viability was determined using the MTT assay as described in Section 2 and was expressed as percentage of control in the mean ± SD of three separate experiments. ^*∗*^ Significant difference as compared to the untreated control group (*p* < 0.05).

We first detected the effect of FZKA decoction on cell growth in human NSCLC cells by MTT assay. As shown in Figures 2(a) and 2(b), FZKA decoction decreased the cell viability in a dose-dependent manner, and the inhibitory concentrations of 50% (IC_50_) observed at 48 h were 17.22 mg/mL and 21.74 mg/mL in A549 and PC9 cells. Similar results were also observed in other NSCLC cell lines (Figure 2(c)).

### 3.3. FZKA Decoction Induced the IGFBP1 mRNA Expression in NSCLC Cells

**Figure 3:**
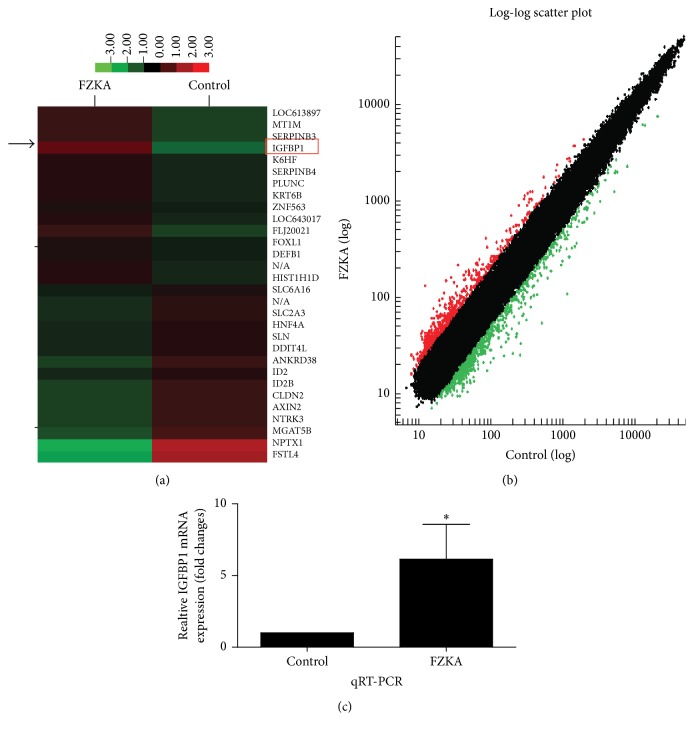
FZKA decoction induced the IGFBP1 mRNA expression in NSCLC cells. (a) Head map compares the fold changes for genes with significantly higher (red) or lower (green) or no expression changes (black) between lung cancer cells in the presence or absence of FZKA decoction for 24 h as determined by NimbleGen Gene chip microarray analysis according to the instruction from the provider. The arrays were scanned with a Roche-NimbleGen MS200 confocal laser scanner, and the obtained images were analyzed using NimbleScan Molecular Annotation System 3.0. (b) Each point on the scatter plot graph represents a gene hybridization signal. The black points represent the ratio that ranged from 0.5 to 2.0, belonging to no different group. The red points represent the ratios that were over 2.0; the green points represent the ratios that were below 0.5. Each row corresponds to a single gene. Color represents different transcript levels, red represents higher gene expression, green represents lower gene expression, and black represents no different gene expression. (c) Total RNA was isolated from A549 cells and a quantitative real-time-PCR assay, as described in Section 2, was used for the detection and quantification of IGFBP1 transcripts. GAPDH was used as internal control. ^*∗*^ Significantly different from untreated cells *p* < 0.05.

In order to explore the regulation of genes affected by FZKA decoction in NSCLC cells, we performed the gene microarray experiments. We showed that FZKA decoction could regulate expression of multiple genes; among them, IGFBP1 is the one, which was highly unregulated by FZKA decoction in A549 cells (Figures 3(a) and 3(b)). This was confirmed by qRT-PCR assays (Figure 3(c)).

### 3.4. FZKA Decoction Increased Phosphorylation of AMPK*α*

**Figure 4:**
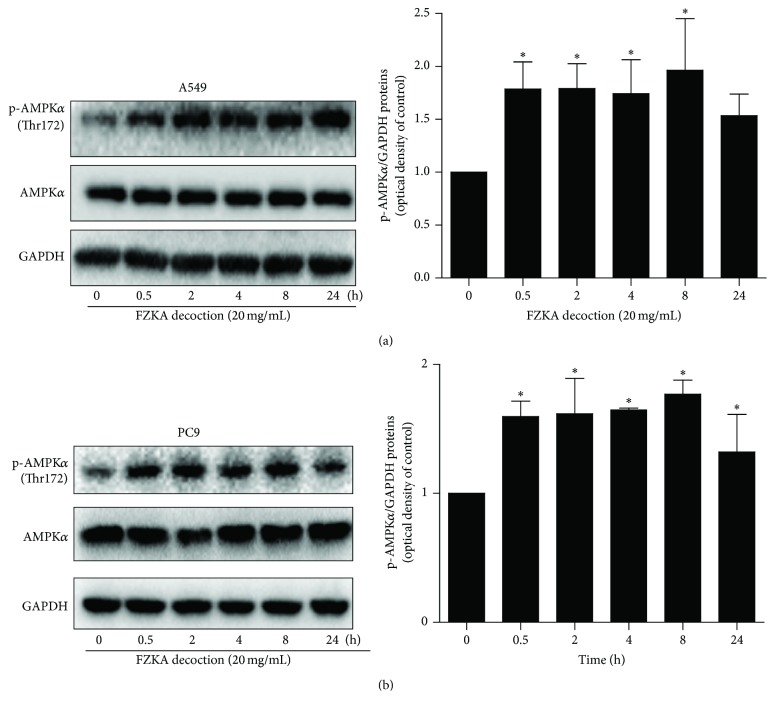
FZKA decoction increased phosphorylation of AMPK*α* in a time-dependent fashion. (a-b) A549 (a) and PC9 (b) cells were exposed to FZKA decoction for up to 24 h, followed by measuring the phosphorylation and protein expression of AMPK*α* by Western blot. The bar graphs represent the mean ± SD of AMPK/GAPDH of three independent experiments. ^*∗*^ Significantly different from untreated cells *p* < 0.05.

In this study, we showed that FZKA decoction increased the phosphorylation of AMPK*α* in a time-dependent fashion, significantly started at 0.5 h, and lasted for up to 24 h in A549 and PC9 cells (Figures 4(a) and 4(b)). Note that the expression of total AMPK*α* proteins had no significant changes after FZKA decoction treatment in A549 and PC9 cells (Figures 4(a) and 4(b)).

### 3.5. FZKA Decoction Induced Protein Levels of IGFBP1 and FOXO3a through Activation of the AMPK*α* Pathway

**Figure 5:**
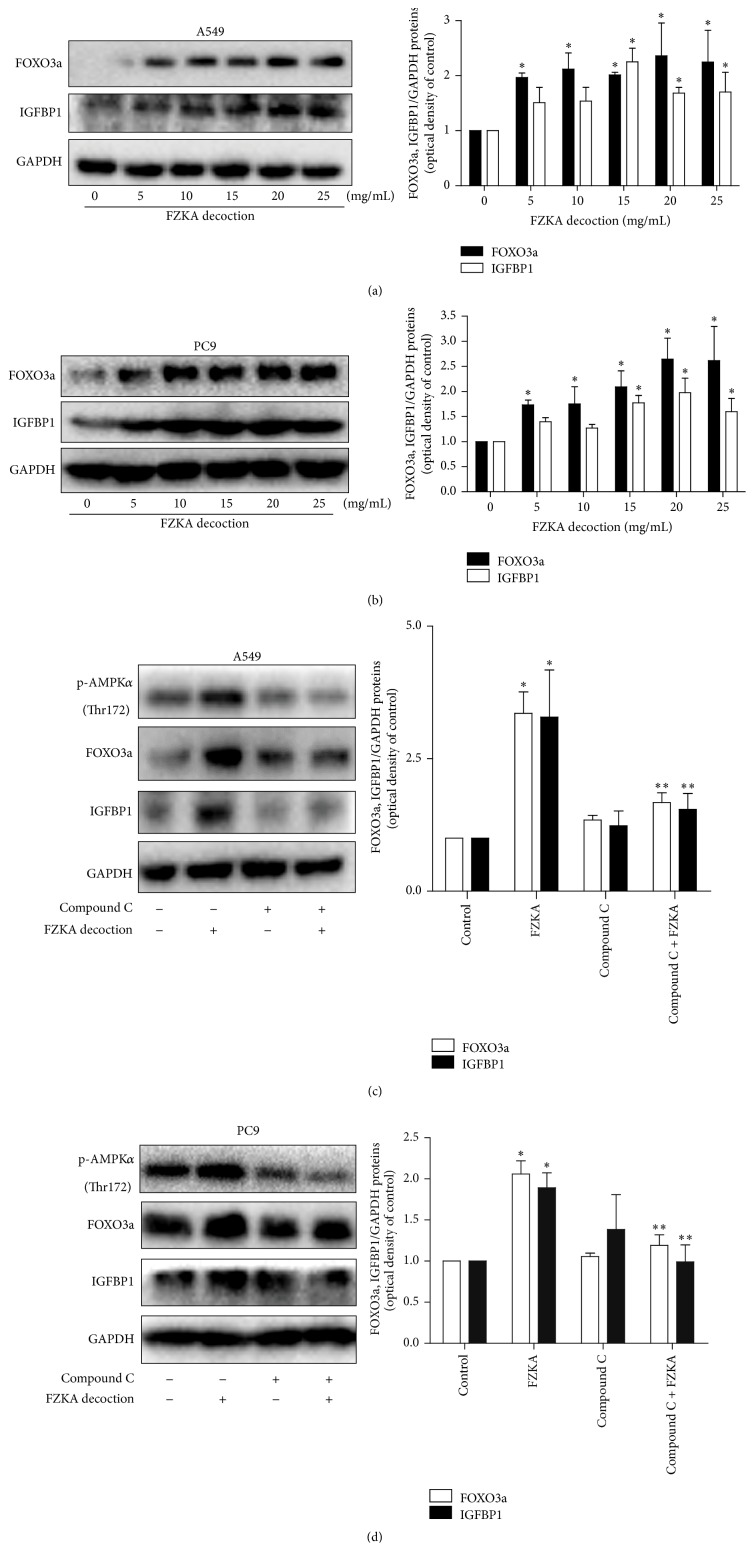
FZKA decoction induced protein levels of IGFBP1 and FOXO3a through the AMPK*α* pathway. (a-b) A549 (a) and PC9 (b) cells were exposed to increased concentration of FZKA decoction for 24 h. Afterwards, the expressions of IGFBP1 and FOXO3a proteins were detected by Western blot. (c-d) A549 (c) and PC9 (d) cells were treated with Compound C (10 *μ*M) for 2 h before exposure of the cells to FZKA decoction (20 mg/mL) for an additional 24 h. Afterwards, the phosphorylation of AMPK*α* and expression of IGFBP1 and FOXO3a proteins were detected by Western blot using antibodies against FOXO3a and IGFBP1. The bar graphs represent the mean ± SD of IGFBP1/GAPDH and FOXO3a/GAPDH of three independent experiments. ^*∗*^ Significant difference from untreated control cells ^*∗∗*^ Significant difference from FZKA decoction treated alone (*p* < 0.05).

Next, we further tested the potential molecular mechanism underlining this effect. We showed that FZKA decoction increased FOXO3a and IGFBP1 proteins expression in a dose-dependent manner in A549 and PC9 cells (Figures 5(a) and 5(b)). Next, we used the special inhibitors of AMPK*α* (Compound C) to pretreated A549 and PC9 cells, to examine the role of this kinase in mediating the effect of FZKA decoction on induction of IGFBP1 and FOXO3a proteins. As shown in Figures 5(c) and 5(d), we found that Compound C abrogated the effect of FZKA decoction on induction of IGFBP1 and FOXO3a proteins expression.

### 3.6. Silencing of IGFBP-1 Reversed the Effect of FZKA Decoction on Cell Growth Inhibition and Abolished the Induction of FOXO3a Protein Expression

**Figure 6:**
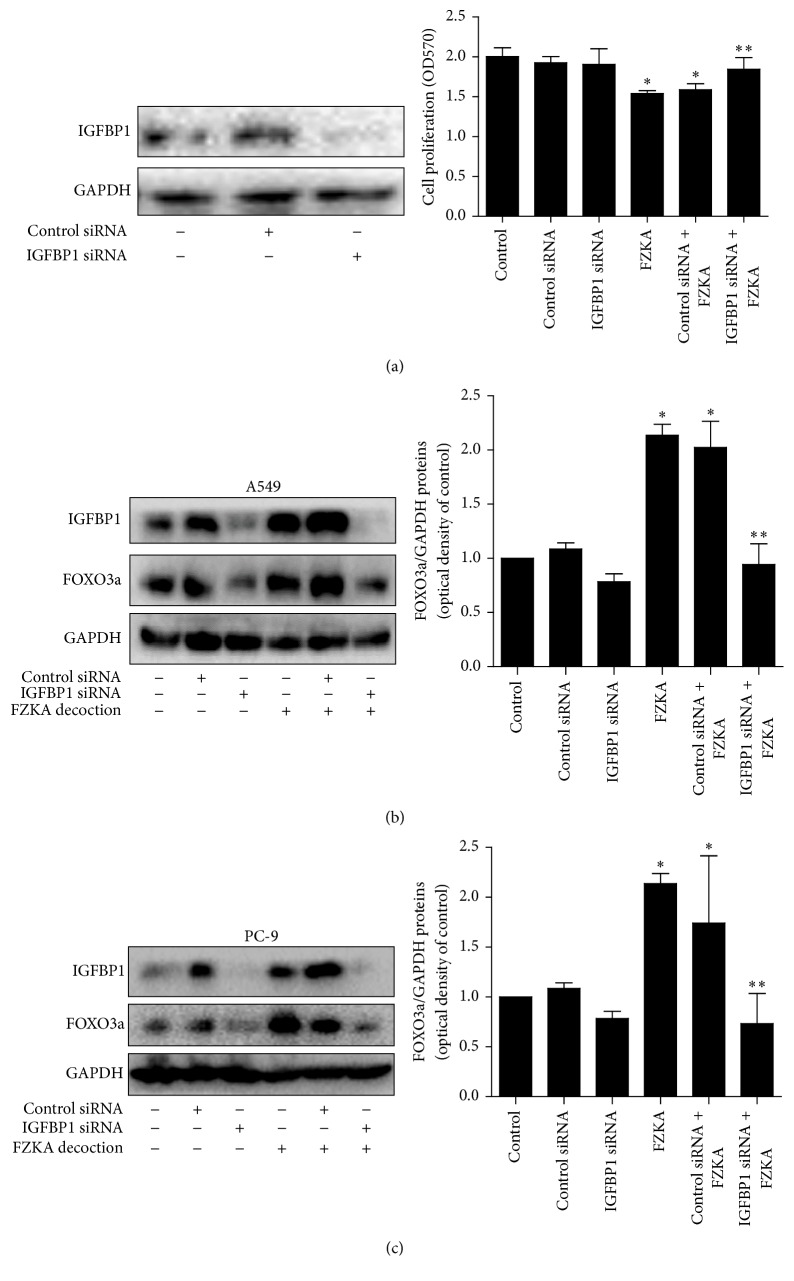
Silencing of IGFBP1 reversed the effect of FZKA decoction on cell growth inhibition and abolished the induction of FOXO3a protein expression. (a) A549 cells were transfected with control or IGFBP1 siRNAs with Lipofectamine RNAiMAX reagent for 24 h, followed by exposure of the cells to FZKA decoction (20 mg/mL) for an additional 24 h. Afterwards, the cells proliferation was detected using MTT assays. The expression of IGFBP1 protein was determined by Western blot. (b-c) A549 (b) and PC9 (c) were transfected with control or IGFBP1 siRNAs with Lipofectamine RNAiMAX reagent for 24 h, followed by exposure of the cells to FZKA decoction (20 mg/mL) for an additional 24 h. Afterwards, the expression of IGFBP1 and FOXO3a proteins was determined by Western blot. The bar graphs represent the mean ± SD of FOXO3a/GAPDH of three independent experiments. ^*∗*^ Significant difference from untreated control cells ^*∗∗*^ Significant difference from FZKA decoction treated alone (*p* < 0.05).

Furthermore, we found that silencing of IGFBP1 by siRNA significantly antagonized the FZKA decoction-inhibited cell growth (Figure 6(a)). In addition, it abolished the FZKA decoction-induced FOXO3a protein expression in A549 and PC9 cells (Figures 6(b) and 6(c)).

### 3.7. While Silencing of FOXO3a Overcame FZKA Decoction-Inhibited Cell Growth, Overexpression of FOXO3a Strengthened FZKA Decoction-Induced IGFBP1 Expression and Phosphorylation of AMPK*α*

**Figure 7:**
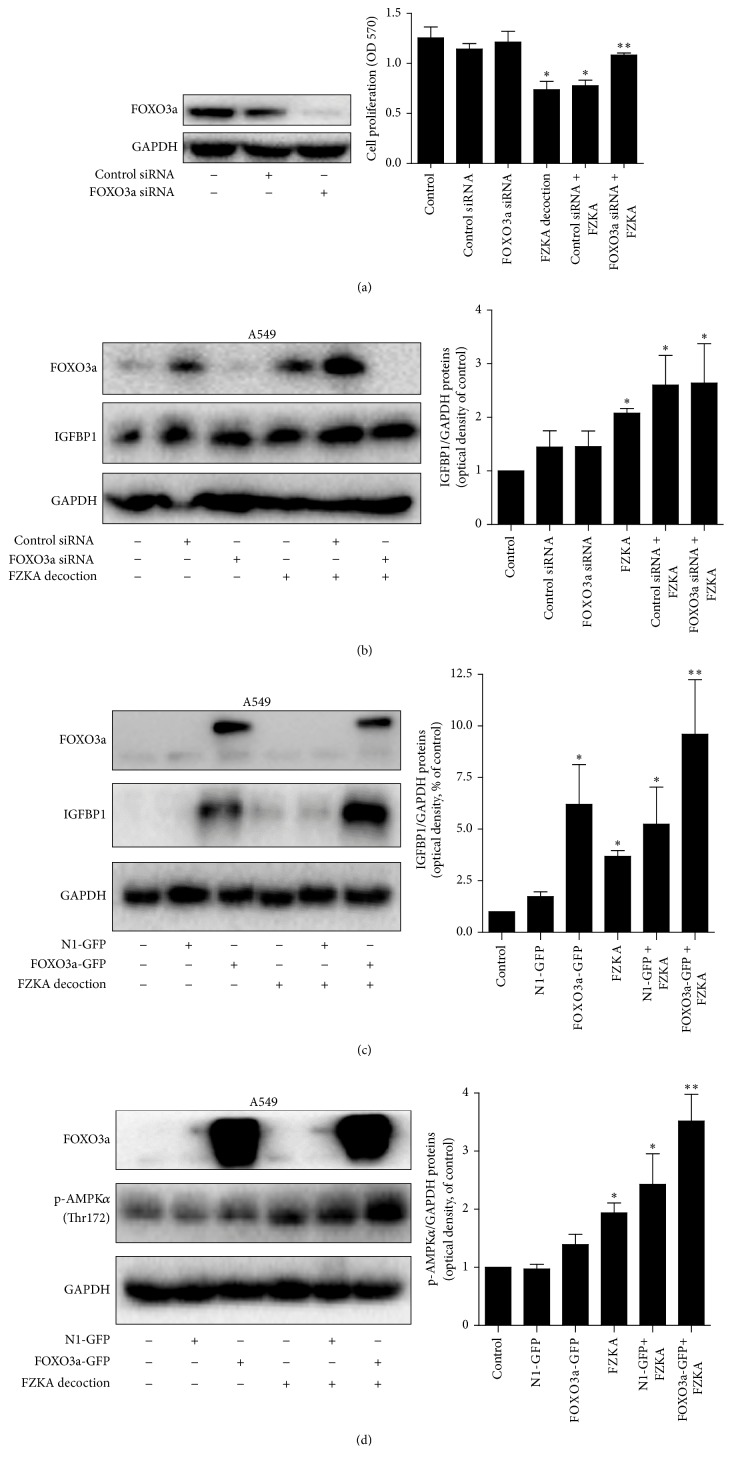
Silencing of FOXO3a overcame FZKA decoction-inhibited cell growth; overexpression of FOXO3a strengthened the effect of FZKA decoction on IGFBP1 expression and phosphorylation of AMPK*α*. (a-b) A549 cells were transfected with control or FOXO3a siRNAs with Lipofectamine RNAiMAX reagent for 24 h, followed by exposure of the cells to FZKA decoction (20 mg/mL) for an additional 24 h. Afterwards, the cells proliferation and expression of FOXO3a and IGFBP1 proteins were detected using MTT assays and Western blot. (c-d) Cells were transfected with control (pEGFP-N1) or FOXO3a (FOXO3a-pEGFP) expression vector for 24 h before exposing the cells to FZKA decoction for an additional 24 h. Afterwards, the expression of FOXO3a and IGFBP1 proteins (c) and phosphorylation of AMPK*α* (d) were detected by Western blot. Data are expressed as a percentage of total cells. Values in bar graphs were given as the mean ± SD from three independent experiments performed in triplicate. ^*∗*^ Significant difference as compared to the untreated control group (*p* < 0.05). ^*∗∗*^ Significant difference from FZKA decoction treated alone (*p* < 0.05).

Moreover, we demonstrated that silencing of FOXO3a by siRNA significantly reversed the effect of FZKA decoction-inhibited cell growth (Figure 7(a)). However, knockdown of FOXO3a had no significant effect on influencing FZKA decoction-regulated IGFBP1 protein expression (Figure 7(b)). On the contrary, exogenous expression of FOXO3a enhanced the effect of FZKA decoction on IGFBP1 protein expression and phosphorylation of AMPK*α* (Figures 7(c) and 7(d)).

### 3.8. The Effect of FZKA Decoction Treatment in Orthotopic Mouse Model

**Figure 8:**
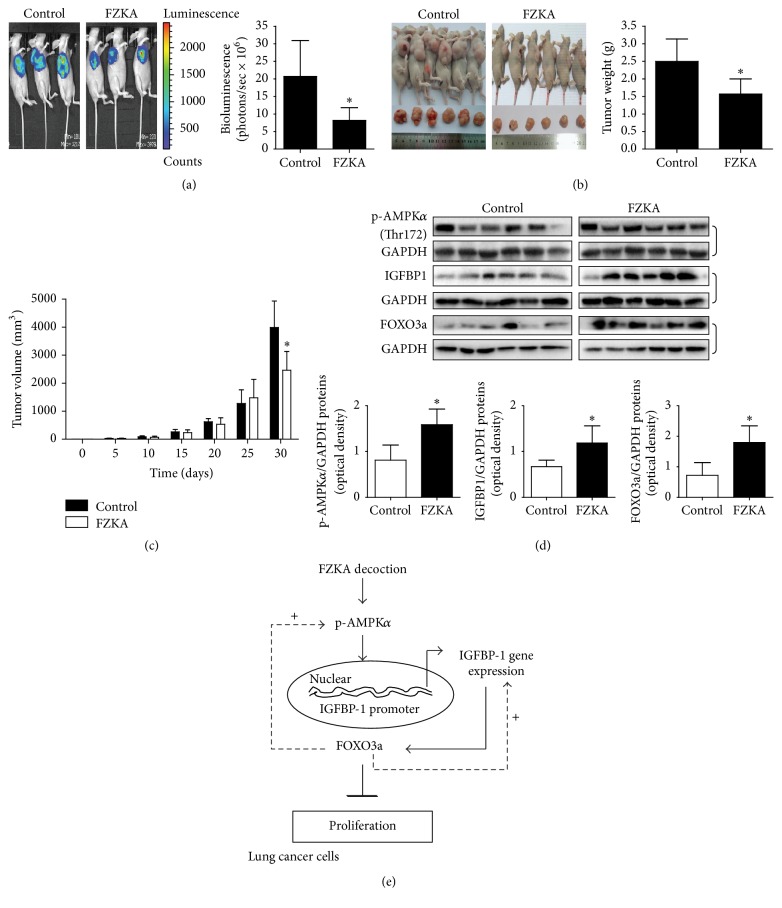
The effect of FZKA decoction treatment in orthotopic mice model. (a) The xenografts were assessed by* in vivo* bioluminescence imaging at the end of the experiments (on day 30). The tumor growth was monitored by injecting luciferin in the mice followed by measuring bioluminescence using IVIS Imaging System. Imaging and quantification of signals were controlled by the acquisition and analysis software living image as described in Section 2. Representative images are shown. ((b) and (c)) The xenografts were harvested on day 30, and the volume and weight of tumors were measured. The bar graphs represented the tumor weight and volume of mice results of mean ± SD. ^*∗*^ Significant difference from untreated control (*p* < 0.05). (d) At the end of the experiments, xenograft tumors were isolated and the corresponding lysates were processed for detecting IGFBP1 and FOXO3a proteins and phosphorylation of AMPK*α* by Western blot. GAPDH was used as loading control. The bar graphs represented the tumor weight and volume of mice results of mean ± SD. ^*∗*^ Significant difference from untreated control group (*p* < 0.05). (e) The schematic diagram shows that FAKA decoction inhibits growth of NSCLC cells through AMPK*α*-mediated increase in FOXO3a and IGFBP1 proteins. Moreover, exogenous expression of FOXO3a feedback strengthened FZKA decoction-induced IGFBP1 and phosphorylation of AMPK*α*. Thus, the reciprocal interplay of IGFBP1 and FOXO3a contributes to the overall response of FAKA decoction.

We also tested therapeutic efficacy of FZKA decoction on the growth of A549-luc cells in nude mouse orthotopic model. We found that, compared to the control group, the FZKA decoction-treated mice showed a significant growth-inhibitory effect as assessed by the Xenogen IVIS200 System (Figure 8(a)). In addition, we noticed a significant reduction of the tumor weight and sizes observed in the FZKA decoction treatment group as compared to that of the control group (Figures 8(b) and 8(c)). Moreover, as expected, we showed that FZKA increased phosphorylation of AMPK*α* and protein expression of FOXO3a and IGFBP1 as compared to that in the control group (Figure 8(d)).

## 4. Discussion

Public interest and demand for complementary and alternative medicine (CAM) services have increased in recent years during the past decade. TCM is an important category of CAM and plays an important role in minimizing disability, protecting cancer patients against suffering from complications, and reducing side effects of conventional treatment [3]. FZKA decoction has been used to treat NSCLC in Guangdong Provincial Hospital of TCM for more than 10 years [7]. However, the mechanisms by which FZKA decoction is improving the therapeutic efficiency against the advanced lung malignancies remains poorly understood.

In this study, we demonstrated cell growth inhibition by FZKA decoction both* in vitro* and* in vivo*, and we confirmed the tumor suppressing effect, which was consistent with the findings from clinical arena [7]. The composition has been reported previously [7]. We demonstrated similar pattern in different batch solutions of FZKA decoction by HPLC; this fair reproducible result indicates a batch to batch consistency. However, we will perform the further quantitative analysis to confirm this. We tested dose ranges in this study, which showed no toxicities* in vitro*; however, more details of the clinical data for the toxicities information need to be well examined and evaluated in the future. All of these implied potential feasibility in control lung cancer growth by FZKA decoction, which was important for the future studies in understanding the complicated networks of signaling pathways and metabolic processes. Although there were important discoveries in this study, there were also some limitations, such as the unknown concentration of FZKA decoction in human blood due to the complexity of chemical components of FZKA, complicated metabolic processes* in vivo*, and relative high cellular drug concentration* in vitro*, which may not reach the human body. This may be the reasons that more substantial effects were found in* in vitro* effects compared to those in* in vivo* human studies. The chemical profiling of main constituents in FZKA decoction using ultra-high pressure liquid chromatography coupled with LTQ Orbitrap mass spectrometry was performed. A qualitative analysis was carried out in both positive and negative ionization modes and accurate mass data were acquired in the full scan analysis, and product ion mass was acquired in the data-dependent MS scan mode (see supplementary data for the details). We believed that the preliminary study will be of great help in developing a high-quality fingerprint for the comprehensive quality control of FZKA decoction. Future studies, such as a series of chemical fingerprints, and more in-depth pharmacokinetic experiments, are required to further identify the truly active components and understand the biological functions of this decoction.

To further explore the molecular mechanism underlining this, we performed the gene array experiments and the results showed that multiple genes were regulated by FAKA decoction. Among those, IGFBP1, metallothionein 1M (MT1M), serpin peptidase inhibitor B3 (SERPINB3), B4 (SERPINB4), zinc finger protein 563 (ZNF563), and forkhead box L1 (FOXL1), among others, were upregulated, whereas hepatocyte nuclear factor 4 (HNF4), neuronal pentraxin 1 (NPTX1), claudin 2, and neurotrophic tyrosine kinase receptor type 3 (NTRKR3), among others, were downregulated. Because of the rare and uncertain conflicting findings for the role of IGFBP1 gene in lung cancer, and also the scope of this study, we only focus on the IGFBP1 gene in the current work. However, we reasoned that future studies are required to elucidate the role and potential mechanisms of those related genes described above that may mediate the antilung cancer effects of FAKA decoction. In this study, we found a remarkable induction of IGFBP1 in the repeated gene array experiments. IGFBP1 interacts with many proteins and plays an important role in transcriptional regulation and DNA damage repair in tumor development, progression, and resistance to treatment [10, 11]. Our results suggested potential tumor suppressor role of IGFBP1 gene and implied that it can be used as a prognostic marker for NSCLC. However, there is limited evidence concerning the role of IGFBP1 expression in NSCLC [25]. Therefore, more studies are required to further prospective investigation for this.

Our results also indicated that the induction and interaction between IGFBP1 and FOXO3a were involved in the inhibitory responses of FAKA decoction on growth of lung cancer cells. We previously demonstrated that induction of FOXO3a was involved in the berberine- and curcumin-inhibited growth and induced apoptosis in NSCLC and nasopharyngeal carcinoma cells [19, 21]. Consistent with this, others also showed similar results indicating the tumor suppressor role of FOXO3a [26, 27]. This implied that FOXO3a represented an attractive therapeutic target in the chemoprevention and possibly in inhibition of progression of human cancers. Moreover, our findings suggested that IGFBP1 could be an upstream of FOXO3a. On the other hand, exogenous expression of FOXO3a positive feedback enhanced IGFBP1 expression. These novel findings suggested that the induction and interplay of these two molecules influenced and contributed to the overall responses of FZKA decoction in inhibition of NSCLC growth. As a known target of FOXO protein, regulation of IGFBP1 promoter activity and gene expression were reported through FOXO-dependent mechanisms [28, 29]. However, scarce information was involved in the interaction between IGFBP1 and FOXO3a. Nevertheless, the detailed mechanism of this interplay in mediating the antitumor activity of FZKA decoction required to be further elucidated.

Intriguingly, our results demonstrated that activation of AMPK*α* was involved in the effect of FAKA decoction on IGFBP1 expression. AMPK plays a central role in the control of cell growth, apoptosis, and autophagy through the regulation of several downstream effectors in cancer cells [30]. The activation of AMPK signaling in mediating the physiopathological responses of cancer cell survival has been shown in other studies [31, 32]. Reports from our study and other studies indicated that activation of AMPK contributed to the increase in IGFBP1 and FOXO3a proteins expression, the decrease in cancer cell growth, and other functions in several cell systems including lung cancer [33–36]. We believed that the feedback regulation loop provided the novel insight into the connection between AMPK signaling and expression of IGFBP1 and also highlighted the tumor suppressor role of AMPK*α* and IGFBP1. Of note, the aforementioned identified mechanisms were limited to cell culture based studies; although we found that similar effects were obtained from the animal studies, the mechanism involved in the effect of FZKA decoction* in vivo* and in human needs to be further determined in the future.

## 5. Conclusion

Our results show that FAKA decoction inhibits growth of NSCLC cells through AMPK*α*-mediated increase in FOXO3a and IGFBP1 proteins. Silencing of IGFBP1 reverses FZKA decoction-increased FOXO3a protein expression. Moreover, while silencing of FOXO3a has no effect on FZKA decoction-induced IGFBP1, exogenous expression of FOXO3a positive feedback strengthens FAKA decoction-induced IGFBP1 protein and phosphorylation of AMPK*α*. Thus, the reciprocal interplay of IGFBP1 and FOXO3a contributes to the overall responses of FAKA decoction. In addition, FZKA decoction inhibits the tumor growth of xenograft nude mice model. This study unveils a novel molecular mechanism by which FAKA decoction controls the growth of human lung cancer cells (Figure 8(e)). More in-depth experiments, such as pharmacokinetic analysis, are required to further identify the active components and understand the biological functions of FZKA decoction.

## Supplementary Material

HPLC grade methanol was purchased from Fisher Chemicals (Fairlawn, NJ, USA). Formic acid was obtained from Guangzhou Chemical Reagent Corporation (Guangzhou, China). Chlorogenic acid, cryptochlorogenic acid, epicatechin and rosmarinic acid were kindly provided by Dr Xiong Li. Accela^TM^ U-HPLC system was purchased from Thermo Fisher Scientific (San Jose, CA, USA). Aglient SB-C18 was ordered from Aglient (Santa Clara, CA, USA). LTQ Orbitrap XL hybrid mass spectrometer was purchased from Thermo Fisher Scientific (San Jose, CA, USA). Data was collected and analyzed with Xcalibur 2.07 (Thermo Scientific).
